# Alzheimer’s disease as a synaptopathy: Evidence for dysfunction of synapses during disease progression

**DOI:** 10.3389/fnsyn.2023.1129036

**Published:** 2023-03-09

**Authors:** Soraya Meftah, Jian Gan

**Affiliations:** ^1^UK Dementia Research Institute, The University of Edinburgh, Edinburgh, United Kingdom; ^2^Centre for Discovery Brain Sciences, The University of Edinburgh, Edinburgh, United Kingdom

**Keywords:** synapse, dysfunction, electrophysiology, oscillations, amyloid, tau, Alzheimer’s disease, synaptic transmission

## Abstract

The synapse has consistently been considered a vulnerable and critical target within Alzheimer’s disease, and synapse loss is, to date, one of the main biological correlates of cognitive decline within Alzheimer’s disease. This occurs prior to neuronal loss with ample evidence that synaptic dysfunction precedes this, in support of the idea that synaptic failure is a crucial stage within disease pathogenesis. The two main pathological hallmarks of Alzheimer’s disease, abnormal aggregates of amyloid or tau proteins, have had demonstrable effects on synaptic physiology in animal and cellular models of Alzheimer’s disease. There is also growing evidence that these two proteins may have a synergistic effect on neurophysiological dysfunction. Here, we review some of the main findings of synaptic alterations in Alzheimer’s disease, and what we know from Alzheimer’s disease animal and cellular models. First, we briefly summarize some of the human evidence to suggest that synapses are altered, including how this relates to network activity. Subsequently, animal and cellular models of Alzheimer’s disease are considered, highlighting mouse models of amyloid and tau pathology and the role these proteins may play in synaptic dysfunction, either in isolation or examining how the two pathologies may interact in dysfunction. This specifically focuses on neurophysiological function and dysfunction observed within these animal models, typically measured using electrophysiology or calcium imaging. Following synaptic dysfunction and loss, it would be impossible to imagine that this would not alter oscillatory activity within the brain. Therefore, this review also discusses how this may underpin some of the aberrant oscillatory patterns seen in animal models of Alzheimer’s disease and human patients. Finally, an overview of some key directions and considerations in the field of synaptic dysfunction in Alzheimer’s disease is covered. This includes current therapeutics that are targeted specifically at synaptic dysfunction, but also methods that modulate activity to rescue aberrant oscillatory patterns. Other important future avenues of note in this field include the role of non-neuronal cell types such as astrocytes and microglia, and mechanisms of dysfunction independent of amyloid and tau in Alzheimer’s disease. The synapse will certainly continue to be an important target within Alzheimer’s disease for the foreseeable future.

## 1. Introduction

The synapse is a highly specialized and complex structure connecting neurons within the brain, which is vital for normal brain function and communication. Synchronized synaptic activity can drive neuronal output and underpin oscillatory activity within the brain, in coordination with intrinsic neuronal properties (Schnitzler and Gross, 2005; Buzsaki, 2006; Fell and Axmacher, 2011). To facilitate synaptic activity, the shape, size, and internal structure of synapses are efficiently constrained but also highly plastic, regulated by activity-dependent changes to the strength or efficacy of synaptic neurotransmission (Konorski, 1948; Hebb, 1949; Engert and Bonhoeffer, 1999; Trachtenberg et al., 2002; Citri and Malenka, 2008; Busse and Smith, 2013). This dynamic process of synaptic regulation is fundamental in learning and memory processes such as long-term plasticity (LTP) and long-term depression (LTD) (Hebb, 1949; Bliss and Lomo, 1973; Bliss and Collingridge, 1993; Citri and Malenka, 2008). Disruption to any of these properties, therefore altering normal synaptic physiology, can lead to widespread neurophysiological dysfunction e.g., seizures (Casillas-Espinosa et al., 2012; Staley, 2015; Du et al., 2019). Brain disorders that have synaptic dysfunction as a key component of their pathogenesis are termed synaptopathies (Li et al., 2003; Lepeta et al., 2016). Therefore, Alzheimer’s disease (AD) has increasingly been considered a synaptopathy (Selkoe, 2002; Jackson et al., 2019; Schirinzi et al., 2020), and this review summarizes some of the evidence of functional changes at the synapse in AD and in animal and cellular models of AD.

## 2. Evidence to suggest synaptic alterations in Alzheimer’s disease

AD is a progressive neurodegenerative disease that affects approximately 1 in 3 people over the age of 65, currently totaling over 50 million people in the world (Prince, 2015; Alzheimer’s Association, 2020). It is the main cause of dementia, which is a symptomatic classifier for alterations in cognitive domains such as memory, language, and reasoning (Alzheimer’s Association, 2020). Along with neurodegeneration, AD has two main neuropathological hallmarks which are formed of abnormal aggregates of amyloid or tau proteins, termed amyloid plaques and neurofibrillary tangles, respectively (Alzheimer Association, 1911; Braak and Braak, 1991). The presence of these two markers appears many years prior to dementia onset in the pathological time course of the disease (Braak et al., 2006; Jack et al., 2009). Interestingly, synapse loss has consistently been observed in AD post-mortem brain tissue, and this loss correlates well with cognitive abnormality, better than other pathological markers in the disease to date (DeKosky and Scheff, 1990; Terry et al., 1991; DeKosky et al., 1996; Selkoe, 2002; Scheff et al., 2006, 2007). Prior to the loss of the synapse, there is overwhelming evidence to suggest these synapses show significant alterations in multiple physiological aspects such as size, shape, and protein expression to name a few examples (Colom-Cadena et al., 2020). Therefore, the synapse has increasingly been considered a key structure within AD and is an important target for cognitive preservation (Selkoe, 2002; Mota et al., 2014; Henstridge et al., 2016; Jackson et al., 2019).

Whilst it is evident that synapses are lost in AD, this loss is not consistent across different synapse subpopulations. Early work in human AD post-mortem tissue across several different brain regions showed a negative correlation between synapse loss and synapse size, with remaining synapses seemingly bigger as synapse loss increases (Adams, 1987; Bertoni-Freddari et al., 1988; Scheff et al., 1990; Scheff and Price, 2003). Synapses in the human hippocampus in AD post-mortem brain tissue were also shown to be larger, with more synapses involved in multisynaptic boutons (Neuman et al., 2015). Using ion beam/scanning electron microscopy in the hippocampus of AD post-mortem brain tissue, there was evidence of an increased proportion of axodendritic asymmetric synapses with a smaller synaptic area (Montero-Crespo et al., 2021). Following studies observed a specific loss of dendritic spine heads in the entorhinal cortex (Domínguez-Álvaro et al., 2019) and also a reduction in inhibitory axodendritic synapses in the hippocampus (Montero-Crespo et al., 2021; Martínez-Serra et al., 2022).

In addition to changes in synapse structure and type, there is also widespread loss and alterations of multiple synaptic markers such as synaptobrevin, synaptophysin, rab 3a, and synaptopodin to name a few (Hatanpää et al., 1999; Reddy et al., 2005). This is not just restricted to excitatory synapses with inhibitory synaptic loss also being evidenced (Kurucu et al., 2022). The development of synaptic markers for PET imaging such as SV2A has allowed for longitudinal visualization of synapses in human patients, and SV2A signal is markedly reduced in AD patients (Chen et al., 2018). Sampling from the cerebrospinal fluid can also reveal markers of synaptic change, with the presence of different synaptic markers such as neurogranin, SNAP25, and GAP43 all elevated within AD patients (Tarawneh et al., 2016; Pereira et al., 2021). Synaptic receptor expression is also reduced in AD. This includes a loss of GluA1 and GluA2 protein expression (Gong et al., 2009; Marcello et al., 2012) and a reduction of GluN1, GluN2A, and GluN2B mRNA and protein levels (Sze et al., 2001; Bi and Sze, 2002; Mishizen-Eberz et al., 2004) in progressed AD post-mortem brain tissue. There are also significant alterations in gene expression by real-time PCR of NMDA, AMPA, and Kainate receptors in AD post-mortem brain tissue (Jacob et al., 2007). Whilst there are changes in excitatory synapses, inhibition was historically thought to be preserved. However, there is increasing evidence that inhibition shows more subtle alterations, and perhaps is critical in AD pathogenesis (Palop and Mucke, 2016; Zott et al., 2018; Ambrad Giovannetti and Fuhrmann, 2019; Bi et al., 2020; Xu et al., 2020). Downregulation of the GABAergic receptors GABA_*A*_ and GABA_*B*_ has been observed in AD post-mortem brain tissue in the middle temporal gyrus in AD (Govindpani et al., 2020). An increased excitatory-to-inhibitory synaptic ratio was observed in the cortex in post-mortem AD brain tissue using fluorescence deconvolution tomography and synaptic membrane microtransplantation (Lauterborn et al., 2021). Apart from changes in glutamatergic and GABAergic systems, there are also changes in many other neurotransmission systems including the endocannabinoid and cholinergic systems (Perry et al., 1987; Mulder et al., 2011; Ju and Tam, 2022).

Isolation of synaptosomes from post-mortem brain tissue has been heavily utilized for research of synapses in AD, and is a valuable tool to assess putative synaptic dysfunction and changes in synaptic protein expression. Some of the earliest work exploring putative synaptic dysfunction assessed isolated synaptosomes from AD post-mortem brain tissue, examining multiple neurotransmission systems (cholinergic, glutamatergic, GABAergic). All systems showed reductions in the uptake of the corresponding neurotransmitter (Rylett et al., 1983; Nordberg and Winblad, 1986; Hardy et al., 1987a,b). This was suggestive of reduced uptake sites or a reduction in the responsivity of synaptosomes from AD patients in each of these systems. Interestingly, the work around the cholinergic system provided some of the earliest evidence for the cholinergic hypothesis of AD (Rylett et al., 1983; Nordberg and Winblad, 1986). Since then, more complex proteomics approaches have also utilized synaptosomes, expanding out from the assessment of neurotransmission or metabolism. Protein marker pathways have highlighted changes in LTP, LTD, and GABA and glutamate receptor signaling pathways in AD (Hesse et al., 2019). The use of SynTOF, which stands for synaptometry by time of flight (SynTOF), allows for multiplexed mass-spectrometry proteomic assessment of synaptosomes. Research using this technique has also found similar results with alterations in pre- and post- synaptic compartments, and inhibitory and excitatory populations (Phongpreecha et al., 2021).

Overall, there are widespread changes in synapse number, structure, synaptic marker expression, receptor expression, protein expression, and putative function, all suggestive of synaptic dysfunction in AD. This would likely lead to further changes in brain function, i.e., network oscillations.

## 3. Synaptic dysfunction and the observed oscillatory abnormality in Alzheimer’s disease

Functional neuronal ensembles are generated by synaptic contacts, coordinated with inhibition and excitation (Buzsaki, 2006). Together, groups of neurons can be linked to fire together to generate changes in the local electrical field, leading to larger-scale synchronous oscillations in the wider area and between other connected brain regions. Oscillations are regulated by inhibitory activity, with precise inputs required for entrainment of neuronal firing (Bartos et al., 2007). Synaptic activity and plasticity help dictate this behavior and therefore, disruption of synapses would lead to disruption of neuronal network activity. Within AD, there are several facets of evidence of abnormal network activity within the disease (Zott et al., 2018; Harris et al., 2020).

Studies using functional MRI have identified a biphasic response in AD in terms of activity, with hyperactivity seen in the earlier phases of the disease, followed by hypoactivity with progression of the disease (Dickerson et al., 2005; O’Brien et al., 2010). PET imaging using fluorine-18 fluorodeoxyglucose, which is an indirect measure of synaptic function/density, showed reduced activity in AD and mild cognitive impairment compared to people without cognitive impairment (Landau et al., 2011; Torosyan et al., 2017). Transcranial magnetic stimulation combined with EEG has been used to evoke synaptically driven cortical activity and measure alterations in connectivity (Nardone et al., 2021). Using this technique, increased evoked activity was associated with worsened cognitive and memory performances (Bagattini et al., 2019). Cortical hyperexcitability was also seen using this method (Casula et al., 2023). Functional assessment of synaptic receptors isolated from post-mortem cortex and hippocampus synaptosomes from AD individuals showed regional differences in the ratio between excitatory and inhibitory currents, which correlated positively with the degree of pathology in the region (Scaduto et al., 2023). Together, this would suggest region- and disease phase- dependent changes in network activity that seem to correlate with disease progression and cognitive alterations (Damoiseaux et al., 2012).

Another often-found phenomenon within AD is a two- to six- fold increased incidence of epileptic seizures in AD patients compared to the general population (Romanelli et al., 1990; Irizarry et al., 2012; Nicastro et al., 2016). Neuronal hyperexcitability or network hypersynchronicity are the main suggested mechanisms for this phenomenon (Nicastro et al., 2016; Tait et al., 2021). This may come from alterations in the regulation of synaptic or neuronal activity, aberrant connectivity (more excitatory, less inhibitory, altered connectivity), or neurodegeneration leading to a loss of balanced excitation and inhibition (Palop and Mucke, 2016; Bi et al., 2020; Giorgi et al., 2020). With the aforementioned changes at the synapse, there would be good reason to suggest that these alterations may relate to seizure emergence in AD.

Other oscillatory disruptions have been observed as well, including disruptions to both gamma and theta band activity (Kitchigina, 2018; Traikapi and Konstantinou, 2021). For example, some studies have observed a loss in both theta and gamma power, whereas other studies have suggested an increase in theta power with a loss of gamma power (Adler et al., 2003; Moretti et al., 2010; Kitchigina, 2018; Traikapi and Konstantinou, 2021). Oscillatory coupling between these two frequencies is also important for learning and memory tasks, and memory transfer (Düzel et al., 2010). This coupling activity was enhanced in AD patients in resting conditions (Wang et al., 2017), but reduced in AD patients during a working memory task (Goodman et al., 2018). In fact, decreased coupling was linked to worsened performance during this working memory task in healthy participants (Goodman et al., 2018). It is therefore evident that there are alterations to multiple oscillatory bands in AD, with disruption to the coordination of this activity as well.

In conclusion, there are many facets of disruption to coordinated neuronal activity, from broad hyper- and hypo- excitability to changes in discreet frequency bands and increased seizure susceptibility. Within the nervous system, changes in homeostatic control, the balance of excitation and inhibition, changes in synaptic strength, and changes in intrinsic neuronal properties may all have an influence on the observed disruption in network activity (Frere and Slutsky, 2018). Animal and cellular models of AD can provide insight into how AD pathology may lead to changes in synaptic function, and the ensuing network alterations.

## 4. The role of amyloid in synaptic dysfunction in Alzheimer’s disease animal and cellular models

The amyloid hypothesis has been a longstanding theme in AD pathogenesis, with the idea that the accumulation of amyloid-β (Aβ) protein aggregates is a key pathological event, based on evidence from causal mutations in early-onset familial AD and neuropathological studies (Chartier-Harlin et al., 1991; Goate et al., 1991; Hardy and Allsop, 1991; Selkoe, 1991; Hardy and Higgins, 1992). Hence, some animal models of AD attempt to recapitulate aspects of Aβ pathology, by either expression of known mutations found within familial AD or by the application of different forms of the amyloid protein. These animal models consistently display functional hyperexcitability, including increased seizure susceptibility and neuronal activity (Palop et al., 2007; Palop and Mucke, 2016; Targa Dias Anastacio et al., 2022). The shift toward excitation in the balance between excitation and inhibition (E/I balance), is one of the main proposed mechanisms for this phenotype in amyloidopathy (Palop et al., 2007). Hyperactivity, which is often seen early in the pathogenesis of AD using functional MRI, and the increased incidence of epilepsy observed in AD, may therefore relate to changes in amyloid leading to a shift in E/I balance (Romanelli et al., 1990; Dickerson et al., 2005; O’Brien et al., 2010; Irizarry et al., 2012; Nicastro et al., 2016). This section summarizes some of the work suggesting altered excitation or inhibition in these animal models. Alterations in E/I balance would also impact plasticity, and within animal models of amyloidopathy there are also persistent alterations in plasticity mechanisms, in particular LTP. This has been thoroughly summarized in many other reviews, and so is not discussed in this review (for examples, see Mesulam, 1999; Marchetti and Marie, 2011; Li and Selkoe, 2020; Schirinzi et al., 2020; Kawabata, 2022).

Genetic mouse models of amyloidopathy typically harbor mutations expressed within familial AD including mutations within the amyloid precursor protein (*APP*) gene, presenilin 1 and 2 (*PS1*, *PS2*) genes, or combinations of the two. These mutations lead to enhanced production or accumulation of Aβ, or increase the ratio between Aβ42 to Aβ40 within the brain (St George-Hyslop et al., 1987; Goate et al., 1991; Scheuner et al., 1996; Bekris et al., 2010). These models generally develop progressive amyloid plaque pathology and can be used as a tool to study the effect of amyloid on synaptic physiology. One such mouse model is the hAPPJ20 model, which overexpresses humanized APP with the Swedish and Indiana mutations linked to familial AD (Mucke et al., 2000). Early work in the hAPPJ20 mouse model in the hippocampus showed increased miniature inhibitory synaptic neurotransmission, both in amplitude and frequency (Palop et al., 2007). Further work in the hippocampus of the same model, showed again an increase in the frequency of miniature inhibitory post-synaptic currents (mIPSCs) and also a reduction in the frequency of miniature excitatory post-synaptic currents (mEPSCs), suggesting a shift in E/I balance. In addition, other alterations in spontaneous and evoked synaptic activity were observed (Roberson et al., 2011). In the cortex decreases in mEPSCs and spontaneous EPSCs (sEPSCs) and spontaneous IPSCs (sIPSCs) has also been observed (Verret et al., 2012). These phenotypes are not just restricted to one mouse model, with similar alterations observed in many other genetic animal models of amyloidopathy (Marchetti and Marie, 2011).

A limitation of the earlier first-generation mouse models of amyloidopathy is that they commonly overexpress amyloid under a non-endogenous promoter, which drives robust pathology but may not be physiologically appropriate (Mckean et al., 2021). Therefore, newer knock-in (KI) mouse models were created with targeted gene-editing in the endogenous genes, resulting in models that express amyloid at physiological levels in a spatially and temporally appropriate manner (Nilsson et al., 2014; Saito et al., 2014; Mckean et al., 2021). The APP*^NL–F^* and APP*^NL–G–F^* are two such models that will be discussed in the following paragraphs. These mouse models express both the Swedish and Iberian mutations within the APP gene, with the inclusion of the Arctic mutation in the APP*^NL–G–F^* model (Nilsson et al., 2014; Saito et al., 2014). Both develop amyloid plaque pathology, with the APP*^NL–G–F^* showing a more rapid and severe phenotype compared to the APP*^NL–F^* mouse model (Nilsson et al., 2014; Saito et al., 2014).

Within the hippocampus in the APP*^NL–G–F^* mouse model, assessment has been performed pre- and post- amyloid plaque emergence. One study has shown synaptic alterations at young ages before plaque pathology, with reductions in sEPSC amplitude in CA1 pyramidal cells and reductions in sEPSC amplitude and frequency in fast-spiking interneurons (Arroyo-García et al., 2021). This was accompanied by a loss of gamma power and impaired gamma coupling of cells. Further changes in synaptic function were also seen at older ages, with decreased sEPSC and sIPSC amplitude seen at 6 months of age. Another study comparing the APP*^NL–F^* mouse model to the APP*^NL–G–F^* mouse model in the hippocampus, only saw changes in sEPSCs (reduction in frequency) at older ages in both models with no changes in the model prior to plaque pathology in sEPSCs. In addition, they also observed a reduction in the paired-pulse ratio (PPR) at ages 9 months or greater in both models with no changes in sIPSCs or in LTP. One study comparing the medial prefrontal cortex to the hippocampus in the APP*^NL–G–F^* mouse model found no changes in synaptic properties in the hippocampus until older (6 months +) ages, where they observed an increased amplitude and frequency of mIPSCs and an increased frequency of mEPSCs (Latif-Hernandez et al., 2020). There were also deficits in LTP at older ages (6 months +). Within the medial prefrontal cortex, there was reduced plasticity from 3 months of age and reduced basal synaptic transmission from 6 months of age (Latif-Hernandez et al., 2020). Interestingly, in the entorhinal cortex in the APP*^NL–F^* mouse model, there were synaptic deficits from an early age (1 month +) (Petrache et al., 2019). This included a reduction in sIPSP amplitude and frequency, with an increase in sEPSP frequency within the model from 1 to 10 months of age. There was also an increase in sEPSP frequency prior to plaque pathology in the model in the entorhinal cortex (Petrache et al., 2019). This was accompanied by other changes in the excitability of pyramidal neurons and parvalbumin-positive (PV) cells. This evidence collectively suggests that the cortex may be more susceptible to changes in synaptic physiology in response to amyloidopathy. This could be related to the structural organization and cellular composition of each of the regions, or the expression and development of pathology in the different regions. In AD patients, there is evidence to support this notion, as there tends to be a stereotypical pattern of pathology progression as described by Braak staging, with evidence of brain region and neuronal subtype specific vulnerability in post-mortem brain tissue (Braak and Braak, 1991; Jack et al., 2009; Fu et al., 2018). Therefore, whilst we cannot directly measure IPSCs and EPSCs in AD patients, the presence of abnormal amyloid accumulation in the brain would likely lead to differences in these measures.

There are also observed alterations in intrinsic properties and dendritic structure in genetic animal models of amyloidopathy, which could also confound or amplify changes in synaptic activity and thus cannot be ignored (Vitale et al., 2021). Whole-cell recordings *in vivo* have revealed a reduction in drive required for burst firing in CA1 neurons in the hippocampus in the APP/PS1 mouse model of amyloidopathy, linked to changes in dendritic structure (Šišková et al., 2014). Studies characterizing intrinsic properties and excitability have also demonstrated conflicting but significant changes in neuronal excitability, such as alterations in hyperpolarization-activated currents and changes in the action potential waveform (Brown et al., 2011; Kerrigan et al., 2014; Tamagnini et al., 2015a; Ohline et al., 2022; Spoleti et al., 2022). All of these factors would also impact synaptic release and function and should be considered when discussing synaptic phenotypes.

Apart from genetic animal models of amyloidopathy, application of various forms of amyloid can alter synaptic activity in a state dependent manner as well. Injection of Aβ oligomers (AβO) into non-human primates led to synapse loss in the injected area (Beckman et al., 2019). AβO injected into the hippocampus of mice led to altered PV- and somatostatin- positive (SST-) neuronal activity in relation to gamma and theta oscillations, respectively, (Chung et al., 2020). There were also reductions in inhibitory plasticity and sIPSCs (Chung et al., 2020). In the same model in the hippocampus, there was disrupted synaptic communication between pyramidal cells and PV cells and alterations in SST- neuronal disinhibition, leading to impairments in gamma oscillogenesis (Park et al., 2020). Alterations in intrinsic excitability can also be seen with the application of AβO to mouse brain slices, which may confound alterations in synaptic function as highlighted previously (Tamagnini et al., 2015b). Molecular mechanisms suggested to underpin some of these changes in physiology due to AβOs includes accumulation of glutamate at the synapse, and dysregulation of glutamate homeostasis leading to increased release of glutamate into the synaptic cleft (Marsh and Alifragis, 2018; Hector and Brouillette, 2021). This may be *via* interactions between Aβ and NMDA receptors (NMDARs), with Aβ seen to impact both the activity of NMDARs leading to increased glutamate in the synaptic cleft or by direct agonistic effects on NMDARs themselves (Arias et al., 1995; Harris et al., 1995; Puzzo et al., 2008; Marsh and Alifragis, 2018). AβOs can also disrupt synaptic vesicle dynamics, including effects on synaptic vesicle docking and fusion, or by altering downstream signaling mechanisms that regulate synaptic vesicle recycling (Russell et al., 2012; Yang et al., 2015; Marsh et al., 2017; Marsh and Alifragis, 2018; Liu A. et al., 2019). These mechanisms may also account for some of the early phenotypes seen in chronic mouse models of amyloidopathy in the absence of plaque pathology.

In addition to oligomeric amyloid, the presence of amyloid plaques can also influence synaptic function. The localization of neurons in relation to plaques can play a role in spine stability and synaptic activity (Dong et al., 2007; Spires-Jones et al., 2007; Busche et al., 2008; Algamal et al., 2022). In the hAPPJ20 mouse model, a reduction in pre-synaptic components is evident in close plaque proximity with no effect of proximity on post-synaptic components or on synapse dynamics (Stephen et al., 2019). In the Tg2576 mouse model, there was increased elimination of dendritic spines close to plaques, without an effect of proximity on synapse dynamics, leading to an overall loss of dendritic spines proximal to plaques (Spires-Jones et al., 2007). In APP23xPS45 mice, hyperactive neurons were exclusively found proximal to plaques, suggested to be due to a decrease in synaptic inhibition (Busche et al., 2008). This effect may be neuronal subtype dependent, as it has been seen that oriens-lacunosum moleculare interneurons, whilst having alterations in activity in amyloidopathy, are unaffected by plaque localization (Schmid et al., 2016). The localization of plaques and progression of pathology should therefore be taken into account when performing studies into synaptic function as this may increase result variability.

Multiple age groups can be used to compare how phenotypes progress or emerge with the progression of amyloid pathology, and reveal how changes in the availability of soluble amyloid oligomers or the presence of plaque pathology may differentially alter physiology. Within the CRND8 mouse model, examined at 2 and 6 months of age in the hippocampus, there were observed alterations in the NMDA:AMPA receptor ratio in the CA1 at both ages, with a reduction seen in the dentate gyrus at the older time point only (Tozzi et al., 2015). This would suggest a differential effect between different brain regions, even if they are close in proximity, which may reflect different levels of pathology or different vulnerabilities of different regions. Field recordings in the hippocampus of APP/PS1 mice at 2–3 months and 8–10 months of age, age groups reflective of pre- and post- plaque pathology onset, respectively, showed LTP alterations at both ages with reduced basal synaptic transmission at the older age only (Gelman et al., 2018). Looking again at the hippocampus in the APP/PS1 mice, synaptic alterations were seen at 8 months but not at 1.5 months, with a suggestion that GABAergic activity may play a role in these alterations (Oyelami et al., 2016). In APPPS1-21 mice, studied at multiple ages, there was increased paired-pulse facilitation at 6 months with decreased LTP seen at 8 and 15 months (Gengler et al., 2010). This suggests that synaptic alterations will depend on the progression of pathology and the brain region vulnerability.

Oscillatory disruption is clear in multiple animal models of amyloidopathy, and this is likely linked to alterations in E/I balance. Specific oscillatory bands of interest include theta, gamma, and sharp wave ripples (SWR), for their roles in learning and memory (Düzel et al., 2010; Joo and Frank, 2018; Herweg et al., 2020). There is evidence for oscillatory disruption across all of the frequency bands specified, with disruption dependent on the model or pathological burden. One common phenotype across animal models of amyloidopathy is a reduction in gamma activity and a slowing of theta, along with alterations in cross-frequency coupling (Mehak et al., 2022). In addition, animal models of amyloidopathy have increased susceptibility to seizures (Palop et al., 2007).

One oscillatory pattern of particular interest is SWRs, which are short bursts of high-frequency electrical activity that are thought to be vital in learning, memory, and memory transfer (Joo and Frank, 2018). During SWRs, phase-locked inhibition is vital for the shaping of SWRs, with PV cells having a critical role in the hippocampus in this process (Gan et al., 2017). An elegant study by Caccavano et al., 2020 attempted to isolate synaptic alterations during SWRs in the 5xFAD mouse model in the hippocampus *in vitro* in brain slices. The incidence of SWRs was increased, with increased SWR amplitude, peak frequency power and associated gamma power, with a reduction in SWR duration and number of oscillatory cycles. During these events, there was increased pyramidal cell activation, with more cells activated during SWR activity. These pyramidal cells during SWRs also received an increased E/I ratio, with a decrease in sIPSC frequency. The authors then dissected out the contribution of different PV interneurons to the contribution of this phenotype. PV basket cells appeared to selectively receive decreased synaptic input and had decreased E/I activity during SWRs. In the same mouse model *in vivo*, there were fewer and shorter SWRs, with evidence that this was due to deficits in inhibitory connectivity to pyramidal cells (Prince et al., 2021). This would suggest that inhibition, and in particular PV cells, may play a role in modulating the deficits seen in these models. This finding seems to be consistent across other animal models of amyloidopathy. Within the APP/PS1 mouse model, PV neuronal projections were increased to CA1 and CA3 pyramidal neurons at an early disease stage, with increased numbers of inhibitory synaptic clusters and synaptic proteins onto pyramidal cells (Hollnagel et al., 2019). There have also been observed differences in SWR coupling with other oscillatory patterns and differences in SWR features in the retrosplenial cortex in the same APP/PS1 mouse model (Yang and Jeong, 2021). Within the APP-KI mouse model, SWR duration was decreased and coordination between the hippocampus and the medial entorhinal cortex was impaired (Funane et al., 2022). In the APP*^NL–G–F^* mouse model, there was a decrease in the power of SWRs with increased synchronicity in PV interneuron activity (Brady et al., 2022). Within an AβO infusion model into the brains of mice, alterations in SWR dynamics were also observed during working memory tasks, with a reduction in SWR occurrence following encoding and recall (Nicole et al., 2016). SWR alterations span multiple animal models of amyloidopathy and this disruption seems in part due to alterations in inhibitory neuronal activity, in particular PV interneurons.

Targeting activity within neuronal subpopulations or modulating receptor activity is a common route used to help restore oscillatory activity in animal models of amyloidopathy. A positive allosteric modulator of GluN2A has been used to enhance synaptic NMDAR activity in the hAPPJ20 mouse model of amyloidopathy (Hanson et al., 2020). This reduced network hypersynchrony and epileptiform discharges seen in the model. Another study within the hAPPJ20 mouse model observed disrupted gamma activity, with decreased sEPSCs, mEPSCs, and sIPSC frequency recorded in pyramidal cells in layer II/III of the parietal cortex (Verret et al., 2012). Further investigation revealed alterations in intrinsic excitability in PV interneurons, which were hypothesized to lead to these observed phenotypes. Upregulation of inhibition by overexpression of Na_V_ 1.1 in the hAPPJ20 mice restored the frequency of sIPSCs and gamma activity, and reduced epileptiform discharges seen in the model. Following work by this group transplanted interneurons that overexpressed Na_V_ 1.1, which led to enhancements of gamma activity and reductions in network hypersynchrony (Martinez-Losa et al., 2018). In another mouse model of amyloidopathy, the APP/PS1 model, there was evidence of hyperactive PV interneurons with altered inhibitory neurotransmission (Hijazi et al., 2020). Chemogenetic inhibition of these PV interneurons rescued these deficits and restored behavioral alterations in the model (Hijazi et al., 2020).

Optogenetic stimulation has also been used to help rescue deficits, presumably by driving synaptic activity, and in particular inhibitory activity to help reduce oscillatory dysfunction. Within the 5xFAD mouse model of amyloidopathy, there was reduced gamma power during SWRs in the hippocampus, which was alleviated by gamma frequency stimulation of PV neuronal activity but not pyramidal cell activity (Iaccarino et al., 2016). This stimulation also seemed to reduce amyloid pathology burden. Within the hAPPJ20 mouse model, observed reductions in gamma oscillations were also rescued with similar gamma frequency stimulation of PV neuronal activity (Etter et al., 2019). Stimulation of SST or PV cells restored reductions in theta and gamma oscillations, respectively, in a mouse model injected with AβO into the brain (Chung et al., 2020). This would suggest that specifically increasing inhibitory activity may balance out excess excitation, whether caused by a loss of inhibition or an increase in excitation.

**FIGURE 1:**
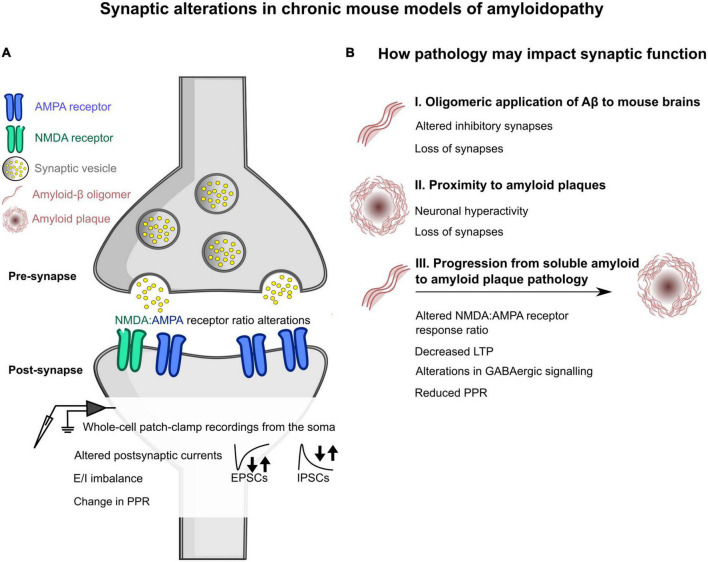
Schematic overview of some of the changes induced by amyloidopathy at the synapse, as seen in chronic mouse models of amyloidopathy. **(A)** General changes in synaptic function at the pre- and post- synapse in chronic mouse models of amyloidopathy. Alterations at the post-synapse in amyloidopathy include a shift in the ratio between AMPA and NMDA receptors expressed at the post-synapse, measured using whole-cell patch-clamp electrophysiological recordings. Whole-cell patch-clamp recordings also showed either increases or decreases in the frequency of spontaneous and miniature excitatory post-synaptic currents (EPSCs) and inhibitory post-synaptic currents, denoted by arrows. The ratio of these two currents (E/I balance) was also skewed in some mouse models. There were also changes in the paired-pulse ratio (PPR), with either increased or decreased ratios measured. **(B)** An outline of how amyloid pathology can impact synaptic function. **(B I)** Changes observed when oligomeric amyloid-β is applied to the brains of mice by microinjection or by topical application to brain slices. This includes altered inhibitory synapses and an overall loss of synapses. **(B II)** How distance to amyloid plaques may alter synapses within chronic mouse models of amyloidopathy. Two phenomena that have been observed are increases in neuronal hyperexcitability when in close proximity to plaques, and an overall loss of synapses as well. **(B III)** How different phenotypes emerge with the progression of pathology in chronic mouse models of amyloidopathy. These experiments investigated changes with age in mouse models that will progressively develop amyloid plaque pathology. Some alterations that were observed at older ages, where plaque pathology is present, included a change in NMDA:AMPA receptor ratio as determined by whole-cell patch-clamp electrophysiology, decreased long term plasticity (LTP), changes in GABAergic signaling, and reduced PPR.

In summary, amyloidopathy alters synaptic function in a variety of manners (Figure 1). Both genetic animal models and also infusion of AβOs into the brains of animals have been shown to disrupt pre- and post- synaptic function, in addition to the impact on network activity. The progression of pathology, i.e., from oligomeric to amyloid plaques, can also have differential effects on function. The idea that E/I balance is altered in amyloidopathy is not a new one, but the advent of newer tools that allow neuronal subtype targeting has allowed the field to probe activity more accurately. Seemingly, inhibitory activity or a loss of inhibitory synaptic function seems to be vital in restoring network activity.

## 5. The role of tau in synaptic dysfunction in Alzheimer’s disease animal and cellular models

Tau is predominantly expressed in the axons of neurons, primarily functions as a microtubule stabilizing protein, and is encoded by the *MAPT* gene (Weingarten et al., 1975; LoPresti et al., 1995; Black et al., 1996; Barbier et al., 2019). Within AD, tau is pathologically hyperphosphorylated leading to dissociation from microtubules, which then forms aggregates termed neurofibrillary tangles, with this hallmark detectable in the brain 2–5 years prior to symptomatic onset (Alzheimer Association, 1911; Bejanin et al., 2017; Vogel et al., 2020). Tau pathology is thought to better reflect changes in cognition, with regional differences in accumulation linking to different behavioral phenotypes and the progression of symptoms (Bejanin et al., 2017). In fact, a subset of neurodegenerative diseases known as tauopathies, are associated with the accumulation of tau without amyloid accumulation, with mutations within the *MAPT* gene leading to familial forms of tauopathy (Hutton et al., 1998; Poorkaj et al., 1998; Spillantini et al., 1998; Kovacs, 2015; Orr et al., 2017).

The role of tau has been less established within synaptic function and dysfunction but is becoming a more indispensable player, and the synapse appears to be particularly vulnerable to tau-mediated disruption (Crimins et al., 2013; Pooler et al., 2014; Jadhav et al., 2015; Tracy and Gan, 2018; Jackson et al., 2019). Hyperphosphorylated tau is preferentially located at synapses in AD, suggesting an influence on synaptic disruption. Specific tau isoform expression within mouse models of AD leads to synapse loss, suggesting subsets of isoforms are synaptotoxic (Eckermann et al., 2007; Sahara et al., 2014; Xia et al., 2015; Schaler et al., 2021). Tau infusion into the brains of mice led to the loss of synapses (Lasagna-Reeves et al., 2011). Chronic mouse models of tauopathy that overexpress familial disease-associated mutations from tauopathies (i.e., Frontotemporal dementia with parkinsonism) show large-scale loss of synapses (Santacruz et al., 2005; Spires et al., 2006; Yoshiyama et al., 2007; Kopeikina et al., 2012). Tau is also released during synaptic activity, and pathological tau spreads across connected neurons *via* synaptic transmission, although the precise mechanism(s) of this seeding is unknown (Pooler et al., 2013; Ahmed et al., 2014). Altogether this would suggest a role for tau in synaptic disruption and loss.

Pre-synaptic dysfunction has been seen when tau is present at the pre-synapse. One group used the squid giant synapse preparation to infuse human full-length “wild-type” tau into the pre-synapse, which led to an acute increase in synaptic transmission followed by a rapid decline in synaptic transmission (Moreno et al., 2011). Another group has performed paired recordings with tau in the intracellular solution in mouse brain slices and found a reduction in synaptic responses in the post-synaptically connected cell, suggesting disruption to pre-synaptic neurotransmitter release (Hill et al., 2019). In addition to tau infusion in the pre-synapse, chronic mouse models of tauopathy have shown alterations in pre-synaptic function including a depletion in the synaptic vesicle pool and an altered probability of neurotransmitter release (Dalby et al., 2014; Polydoro et al., 2014; Decker et al., 2015). Some of the mechanisms implicated in tau-associated pre-synaptic dysfunction include tau binding to synaptic vesicles, increases in cytosolic calcium, and disrupted mitochondrial trafficking leading to disrupted synaptic transmission (Decker et al., 2015; Moreno et al., 2016; Zhou et al., 2017; McInnes et al., 2018; Wu et al., 2021).

Post-synaptic disruption is also evident in multiple animal and cellular models of tauopathy, with conflicting impact ranging from widespread loss and dysfunction to preserved excitatory neurotransmission (Crimins et al., 2013). A loss of mEPSCs was linked to impaired glutamate receptor trafficking in an *in vitro* cellular model of tauopathy (Hoover et al., 2010). As tau has been suggested to interact with NMDARs *via* Fyn kinase, this has become a particular focal point for a mechanism of excitatory neurotransmission alterations (Ittner et al., 2010; Mondragón-Rodríguez et al., 2012; Miyamoto et al., 2017). In addition, glutamate excitotoxicity mediated by NMDARs, is a suggested mechanism of neurodegeneration in AD (Mota et al., 2014; Wang and Reddy, 2017; Liu J. et al., 2019). Other post-synaptic receptors are also altered, with differential phosphorylation of tau differentially reducing AMPA receptor (AMPAR) activity in *in vitro* cultured neurons (Teravskis et al., 2021). Conversely, increased putative AMPAR-mediated activity was observed in the chronic rTg4510 mouse model in both the hippocampus and cortex at a stage of progressed pathology and neurodegeneration (Crimins et al., 2011, 2012; Dalby et al., 2014). Some molecular mechanisms by which tau may lead to post-synaptic disruption includes the promotion of AMPAR endocytosis and LTD *via* PICK1, alterations in glutamate receptor trafficking, and impaired plasticity mechanisms in LTP and LTD (Yagishita et al., 2015; Wu et al., 2021).

Apart from the pre-synapse and post-synapse as individual components, it is clear that tau pathology also alters synaptic stability and plasticity. Aberrant dendritic spine dynamics and activity has been observed in the chronic rTg4510 mouse model of tauopathy (Jackson et al., 2017, 2020). The structure of synapses *in vivo* is altered in the P301S mouse model of tauopathy in the cortex with a shift seen toward mushroom spines and a loss of thin spines (Hoffmann et al., 2013), whereas the converse is seen in the cortex of the rTg4510 mouse model of chronic tauopathy with a shift toward more immature synaptic structures such as filopodia and a loss of more mature mushroom spines (Crimins et al., 2011). In regards to plasticity, intracellular infusion of tau into neurons from mouse brain slices whilst recordings from the post-synaptic cell led to a blockage of short-term plasticity (STP) and LTP (Hill et al., 2019). Extracellular application of tau oligomers, applied either by incubation on acute mouse brain slices or infused into the brains of mice, also leads to the impairment of LTP (Fá et al., 2016; Goate et al., 2017). Chronic mouse models of tauopathy have also shown alterations in plasticity, in both long- and short- term mechanisms (Sydow et al., 2011; Booth et al., 2014, 2016; Gelman et al., 2018). One chronic model of tauopathy, the Tau35 mouse model, showed normal LTP with increased STP at 10 Hz stimulation frequencies, suggesting different models may show different plasticity alterations dependent on tau isoform expression (Tamagnini et al., 2017). These changes in plasticity are not just limited to *in vitro* assays, as disrupted short- and long- term visual plasticity has also been observed *in vivo* in the rTg4510 mouse model of tauopathy (Papanikolaou et al., 2022).

Within animal models of tauopathy, there are observed alterations in network activity. Tau seems to be able to influence network activity *via* synaptic connectivity. One powerful piece of evidence for this is the spreading of tau across synapses termed “seeding,” which seems to sequentially impact activity and connectivity (Ahmed et al., 2014; Ahnaou et al., 2017; Marinković et al., 2019). Infusion of tau into a region of low pathology in the P301S mouse model of tauopathy led to a reduction in spontaneous neuronal activity (Marinković et al., 2019), and using the same paradigm in the P301L mouse model led to alterations in oscillations and synchronicity between regions suggesting disruption of coordinated activity (Ahnaou et al., 2017). Chronic tauopathy animal models without tau infusion also show altered network activity and connectivity. Within the TauRD mice, which express pro-aggregant or anti-aggregant mutations in the human tau repeat domain, functional MRI connectivity was significantly reduced (Green et al., 2019). An elegant study comparing *in vivo* intracellular and extracellular recordings within the neocortex observed longer down states and altered firing rates of intracellular neuronal activity, linked to reduced frequency of slow-wave oscillations observed at the network level in the rTg4510 mouse model of tauopathy (Menkes-Caspi et al., 2015). “Silent” cells are also observed in the chronic rTg4510 mouse model of tauopathy, classified by a loss of intracellular Ca^2+^ transients, suggesting a reduction in overall neuronal and thus network activity (Busche et al., 2019). Overall, the influence of tau may result in changes or a decrease in neuronal and network activity. AD displays biphasic functional disruption in network activity with hyperactivity followed by hypoactivity, with the latter potentially associated with the development and advancement of tau pathology within the brain (Dickerson et al., 2005; O’Brien et al., 2010; Harris et al., 2020). Interestingly, this network disruption seems to be specific to certain networks, as some show resistance to tauopathy-induced disruption compared to others. Within the rTg4510 mouse model, visual cortical network activity as measured by intracellular Ca^2+^ transients was functionally intact (Kuchibhotla et al., 2014). Functionality of head direction cells also seemed unimpaired in the entorhinal cortex, in contrast to deficits in grid cell activity and local network coordination in the same brain region in the rTg4510 mouse model (Ridler et al., 2020). This may explain why some behaviors and function is preserved in human AD, whilst others show impairment and loss.

**FIGURE 2:**
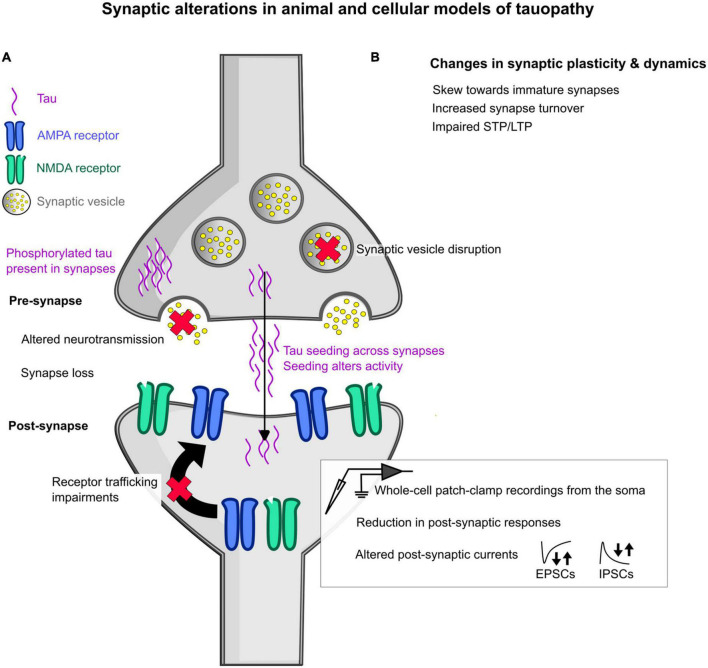
Schematic overview of some of the changes induced by tauopathy in animal and cellular models at the synapse. **(A)** General changes in synaptic function at the pre- and post- synapse in animal models of tauopathy. In the pre-synapse, synaptic vesicle disruption has been observed, as denoted by a red cross over the synaptic vesicle. Altered neurotransmission has also been seen, denoted by a red cross over the neurotransmitters shown as yellow dots. The detection of mislocalized phosphorylated tau has been seen at both the pre- and post-synapse, highlighted as purple waves in each synaptic space. Tau is also thought to seed across synapses by multiple hypothesized mechanisms, with this seeding shown to alter activity in the brain. At the post-synapse, glutamatergic receptor trafficking is impaired showing a loss of receptors at the post-synaptic domain. In addition, measures using whole-cell patch-clamp electrophysiological recordings revealed reductions in post-synaptic responses to stimulation and increased or decreased frequency of spontaneous and miniature excitatory post-synaptic currents (EPSCs) and inhibitory post-synaptic currents (IPSCs), denoted by the arrows. **(B)** Changes that have been observed in synapse plasticity and dynamics include a skew in synapse shape toward “immature” synapses, increased synapse turnover, and impairments in short-term and long-term plasticity (STP and LTP, respectively).

Overall, the evidence of how animal and cellular models of tauopathy can influence synaptic activity is understudied but rapidly growing compared to amyloidopathy (Figure 2). Whilst it seems that tau can have a pathological influence on synaptic function, the mechanistic timeline and the cause of variability between different synapses or connections is still unclear.

## 6. Interaction between amyloid and tau at the synapse?

Recent evidence has shown that animal and cellular models which express both amyloid and tau pathology may have a synergistic effect on physiology, leading to investigations on how these two pathological proteins may interact to impact function (Forner et al., 2017; Harris et al., 2020). In particular, the synapse has been highlighted as a node to integrate such dysfunction (Crimins et al., 2013; Spires-Jones and Hyman, 2014).

There is limited work looking at synaptic function in animal and cellular models with dual amyloid and tau pathology. In APP/PS1 mice crossed with Tau knock-out (KO) mice or Tg21221 mice which express human tau only, there was observed downregulation of synaptic genes for pathways such as for LTP, long-term depression (LTD), and glutamate receptor signaling, seen in the combined model compared to the respective controls (Pickett et al., 2019). One study examined intracellular Ca^2+^ transients in a crossed model between the APP/PS1 mouse model of amyloidopathy and the rTg4510 mouse model of tauopathy and observed reduced neuronal activity, compared to the individual amyloid and tau mouse models which showed hyperexcitability and neuronal silencing, respectively (Busche et al., 2019). This study concluded that the two pathologies have differential effects on function, but that tauopathy may dominate the overall physiological phenotype. This seems plausible as this would better reflect the pathological time course of AD in relation to cognitive alterations, as tau pathology onset occurs much closer to symptomatic onset than amyloid pathology (Braak and Braak, 1991; Jack et al., 2009). AβO incubation applied to cell cultures was shown to lead to tau missorting, a loss of microtubules, and has also been shown to phosphorylate tau in a GSK-3-dependent manner (Shipton et al., 2011; Zempel and Mandelkow, 2012). There are many suggested mechanisms for how Aβ and tau may interact on a molecular level, which could impact and influence function. This includes Aβ accelerating the phosphorylation of tau *via* kinases, and Aβ can also promote tau oligomerization and cleavage *via* GSK-3β and multiple caspases, respectively (Zheng et al., 2002; Gamblin et al., 2003; Zhang et al., 2021). The Aβ core of aggregates may itself interact with tau directly to promote tau aggregation (Griner et al., 2019). The presence of dendritic tau can mediate and augment Aβ toxicity *via* Fyn and other tau dependent signaling cascades in dendritic spines (Ittner et al., 2010; Polanco et al., 2018). Crosstalk between the two proteins has also been shown to interface at mitochondria, which would directly impact energy availability at the synapse and consequently function (Rhein et al., 2009; Polanco et al., 2018). Overall, there is an idea that amyloid may prime the environment for tau effects, and also work together downstream in a coordinated cascade to influence function.

Utilization of the TauKO mice has allowed for the role of tau in amyloid pathophysiology to be dissected, by crossing these mice with amyloid mouse models. One study used hAPP mice crossbred with TauKO mice, with this model showing rescued cognitive deficits and reduced seizure severity compared to the amyloid or tau mouse lines alone (Roberson et al., 2007). A following study used the hAPPJ20 mice crossed with TauKO mice, studying the dentate gyrus for alterations in synaptic physiology. Within the hAPPJ20 mice, there were large-scale alterations at the synaptic level impacting basal synaptic activity, E/I balance, and evoked activity, with these differences normalized in the hAPPJ20/TauKO mice (Roberson et al., 2011). AβO applied to the brain slices of TauKO mice prevented AβO induced LTP deficits (Shipton et al., 2011). This would suggest that lowering physiological tau may have a protective effect on the impact of amyloid pathology and that the two may interact to lead to pathophysiological phenotypes. Alternatively, a more recent study used the TauKO mice crossed with the TgAPP mouse model of amyloidopathy, in addition to the oligomeric application of tau or amyloid within the Tau KO mice, look at the effect of endogenous tau on synaptic physiology (Puzzo et al., 2020). This showed evidence that the TauKO mice themselves may have altered basal synaptic physiology, and tau was not required for the induction of AβO impairments. This work would suggest that perhaps the two pathologies work in parallel and not in coordination, and there are some theories that propose that amyloid and tau pathology work separately but are linked by upstream drivers and downstream pathways (Small and Duff, 2008).

Overall, the interaction between amyloid and tau and how this may alter synaptic physiology is understudied and currently reveals conflicting results. This suggests that these proteins may exert their effects by both parallel and interacting pathways. Although this may be difficult to investigate and can add extra challenges, more work is needed to understand and characterize how tau and amyloid disruption may alter physiology together and separately within AD.

## 7. Discussion and future directions

**FIGURE 3:**
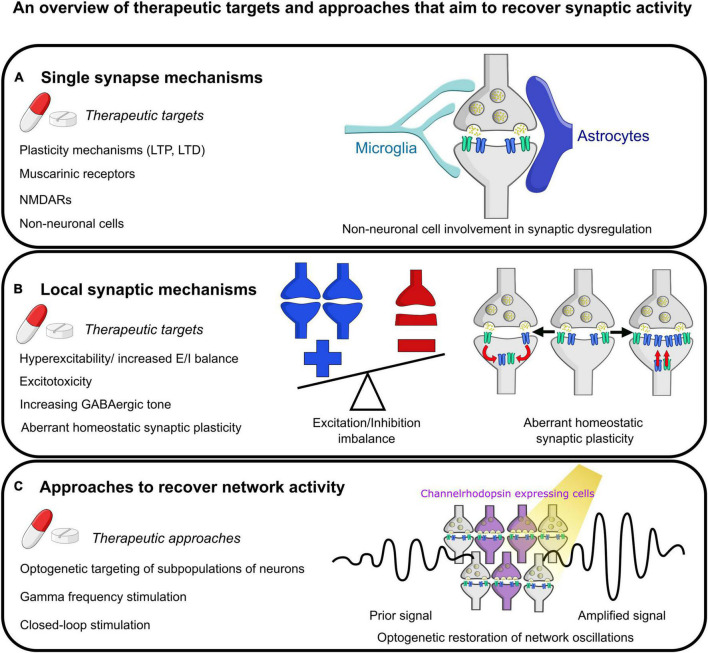
Summary of some future therapeutic targets aimed at restoring normal synaptic physiology. **(A)** Mechanisms targeting individual synapses. This includes targeting short- and long- term plasticity (STP and LTP, respectively) at the synapse, modulation of muscarinic receptor and NMDA receptor activity, and targeting aberrant activity of non-neuronal cells such as microglia and astrocytes on synapses. **(B)** Therapeutic mechanisms at the local synaptic level. This includes reducing hyperexcitability or restoring E/I imbalance, preventing excitotoxicity, increasing GABAergic tone, and evaluating the effect of aberrant homeostatic synaptic plasticity. The example images show an imbalance of excitation and inhibition with more excitation as has been described in the literature. Next shows one mechanism of homeostatic synaptic plasticity, synaptic scaling, with either abnormal increases or decreases in synaptic strength by altering the number of receptors present at the post-synaptic density. **(C)** Therapeutic mechanisms that could be utilized to rescue network activity including optogenetic modulation of the activity of subpopulations of neurons (as shown in the pictogram), stimulation of neurons at gamma-band frequency, and more sophisticated closed-loop network modulation to attempt to recover activity to normal physiological set points following deviations.

The synapse is a critical structure in AD, and recovering synaptic function has been and continues to be a major target for therapeutics (Figure 3). The majority of approved therapeutics for AD target synaptic function. Donepezil, Rivastigmine and Galantamine are all cholinesterase inhibitors, and Memantine is an NMDAR antagonist (Athar et al., 2021; Nguyen et al., 2021; Ju and Tam, 2022). However, these only provide some mild symptomatic relief and do little against the progression of the disease. Interestingly, there is evidence that these drugs can modulate theta and gamma oscillations within AD (Isla et al., 2021). Future therapeutics continue to target the synapse, with drugs aimed at improving synaptic function in a multitude of aspects such as enhancing LTP, decreasing LTD, and modulating NMDARs and muscarinic receptors (Jackson et al., 2019; Yu et al., 2021; Ju and Tam, 2022). E/I imbalance is another therapeutic target, with therapeutics designed to reduce excess toxicity or increase GABAergic tone (Calvo-Flores Guzmán et al., 2018; Ju and Tam, 2022).

Other therapeutic approaches that are being pursued include modulation and enhancement of oscillations (Chan et al., 2021a; Mirzayi et al., 2022). Deep brain stimulation, transcranial magnetic stimulation, and transcranial electrical stimulation are all being explored as ways of modulating activity in AD with some modest improvements seen with these methods (Laxton et al., 2010; Yu et al., 2021). However, these methods are quite generalized and broad ways to drive activity due to the technical limitations of these techniques. Some more refined approaches includes the use of optogenetics to modulate activity of specific neuronal subtypes with plenty of evidence from animal models of AD of therapeutic efficacy, albeit with large translational limitations to get to the clinic (Giovannetti et al., 2018; Etter et al., 2019; Park et al., 2020; Mirzayi et al., 2022). In addition to targeting subpopulations of neurons, enhancing specific oscillatory band activity has shown some promise. Driving gamma-band oscillatory activity within animal models of AD has shown intriguing results in the rescue of memory deficits and also reductions in pathology (Iaccarino et al., 2016; Martorell et al., 2019; Adaikkan and Tsai, 2020). So much so, this work has progressed into phase 2 studies, showing some benefits in patients with AD (Chan et al., 2021b; Cimenser et al., 2021). Closed-loop stimulation is a refined method to modulate activity in real-time by measuring deviations away from the desired set point, in this review meaning activity of the brain, and providing feedback to alter the delivered stimulus. Closed-loop optogenetics uses closed-loop control theory to modulate optogenetic stimulation to a set activity point measured from brain activity (Grosenick et al., 2015). The idea of closed-loop stimulation, and in particular closed-loop optogenetics for more precise activity modulation and stimulation is also an appealing avenue for future therapeutics (Senova et al., 2018; Chan et al., 2021a).

One factor to consider is whether all changes in synaptic function are harmful, where synapses may alter their activity or sensitivity to help restore balance to network activity. Complex compensatory mechanisms such as changes in synapse scaling and metaplasticity may be in play to rescue existing synaptic deficits, preserving cognitive function (Abuhassan et al., 2014; Baazaoui et al., 2017; Styr and Slutsky, 2018; Jackson et al., 2019). Therefore, targeting some of these key mechanisms may lead to worsened impairments (Styr and Slutsky, 2018). Increasing synaptic LTP on a global level for example may lead to increased activity in regions that are already hyperplastic, leading to worsening of function. There may also be a failure of homeostatic control mechanisms to regulate function, which may have downstream consequences in other regulatory pathways (Styr and Slutsky, 2018). Specific targeting of subregions and subpopulations of neurons and modulating their specific activity is a critical point of consideration for further therapeutic design.

Whilst this review has primarily focused on the direct impact of amyloid and tau on synapses, there are many other potential contributors to synaptic dysfunction in AD. For instance, before the onset of clinical symptoms, there are a number of other physiological changes that may occur independently or due to amyloid and tau pathology. One such example is the impairment of cerebral blood flow and other neurovascular changes which has been linked to the early stages of AD, even prior to Aβ and tau accumulation in the brain (Sweeney et al., 2018). Changes in blood flow and alterations in the cells that regulate the blood brain barrier (e.g., pericytes, endothelial cells, astrocytes) would impact synapses by potentially uncoupling neuronal energy demand to provision of increased localized blood flow and nutrients, and by the loss of glymphatic clearance of waste (Zlokovic, 2008; Venkat et al., 2016; Sweeney et al., 2018).

Additionally, changes in white matter are considered another early pathological change in AD, and Aβ and tau can also lead to damage of white matter (Bartzokis, 2011; Nasrabady et al., 2018). This has also been seen in mouse models of AD such as the 3xTg-AD mice, where myelin and oligodendrocyte disruption was evident prior to the emergence of tau or amyloid pathology (Desai et al., 2009). AβO injection in the brains of mice led to myelin damage and oligodendrocyte toxicity, suggesting a direct interaction between Aβ and myelin integrity (Jantaratnotai et al., 2003). This may be *via* overactivation of NMDARs, which can lead to oligodendrocyte death and myelin destruction (Salter and Fern, 2005; Micu et al., 2006). In direct relation to synapses, the myelination-based hypothesis of AD suggests that synapse loss and synaptic change may be in response to loss of myelin in an attempt to preserve myelin integrity in the brain (Bartzokis, 2011).

Other mechanisms may also include abnormal Ca^2+^ signaling and mitochondrial dysfunction. Disruption in Ca^2+^ signaling in the endoplasmic reticulum was associated with synaptic destabilization and a loss of synaptic markers and neuronal activity, in the PS1-M146V mouse model of AD which is independent of amyloid or tau pathology (Sun et al., 2014; Zou et al., 2022). Synaptic mitochondrial dysfunction, whether due to direct effects of Aβ or tau on mitochondria or indirectly, is also another potential candidate (Hauptmann et al., 2006; Wang et al., 2020). For example, oligomeric tau infusion into the brains of mice leads to mitochondrial and synaptic dysfunction, and axonal trafficking and distribution of mitochondria can also be disrupted in animal models of AD (Kopeikina et al., 2011; Lasagna-Reeves et al., 2011; Wang et al., 2020).

Other non-neuronal cell types also have to be appreciated when discussing the synapse, as the evidence for their role in synaptic function and regulation has grown considerably. Glial cells, and in particular astrocytes and microglia, are two key cell types to consider in this space. Indeed, it has been shown that both astrocytes and microglia contain synaptic proteins in AD post-mortem brain tissue, suggesting abnormal engulfment or uptake of synapses by these cells (Tzioras et al., 2021). The injection of AβOs into non-human primates led to increased PSD95 presence in microglia (Beckman et al., 2019). With the role of microglia in synaptic pruning and engulfment, there is evidence for an abnormal promotion of microglial phagocytosis of synapses in AD, perhaps from tagging of synapses with oligomers or classical complement pathway dysregulation (Rajendran and Paolicelli, 2018; Bartels et al., 2020). AD risk genes also include immune-related genes such as *TREM2*, which is expressed in microglia (Rajendran and Paolicelli, 2018; Bartels et al., 2020). The other cell type mentioned, astrocytes, are a critical component of the tripartite synapse, which has an intimate role in synaptic function and therefore a possible role in dysfunction (Araque et al., 1999). Astrocytes both monitor and respond to synaptic activity, which provides feedback to fine-tune synaptic activity (Araque et al., 1999; Ventura and Harris, 1999; Perea et al., 2009). They can also rectify synaptic overspill and communicate with local vasculature to help regulate neuronal energy consumption (Perea et al., 2009; Chung et al., 2015). Astrocytes also perform synaptic pruning and are involved in phagocytosis (Chung et al., 2013; Byun and Chung, 2018). *APOE* genotype is one of the major genetic risk factors in AD, and astrocytes are the main source of endogenous APOE in the brain in physiological conditions (Corder et al., 1993; Saunders et al., 1993; Strittmatter et al., 1993; Huang et al., 2004). Interestingly, it has been shown that the rate of synaptic pruning by astrocytes can be dependent on the *APOE* allele expressed (Chung et al., 2016). Astrocytes may also have a role in Aβ uptake and degradation *via* APOE (Verghese et al., 2013). Microglia and astrocytes may therefore have a key role in synaptic dysfunction and loss in AD through alterations in phagocytosis, reduced support of synapses, and by other indirect effects on synaptic function (Piccioni et al., 2021; Hulshof et al., 2022). Other glial cells such as oligodendrocytes may also contribute to synaptic dysfunction, by alteration of myelination and due to toxicity induced by Aβ and tau, leading to loss of function which may disrupt signaling (Bartzokis, 2011; Butt et al., 2019).

## 8. Conclusion

The synapse is an ever-critical structure in AD, with evidence in both human patients and animal and cellular models that alterations in synaptic function is occurring prior to synapse loss. There is promising work to progress some of the synaptic mechanisms underpinning oscillatory disruption, particularly in animal and cellular models of amyloidopathy. Whilst we have made progress in understanding this dysfunction, more needs to be done to understand how changes in function link to network activity to help with translatability to AD.

## Author contributions

JG acquired funding for this work. SM wrote the first draft of this manuscript. Both authors conceptualized the initial review outline, reviewed, edited, and finalized the submitted manuscript, contributed to the revision of this article, and approved the submitted version.
